# Cultivation and Immortalization of Human B-Cells Producing a Human Monoclonal IgM Antibody Binding to MDA-LDL: Further Evidence for Formation of Atherogenic MDA-LDL Adducts in Humans *In Vivo*

**DOI:** 10.1155/2017/6047142

**Published:** 2017-11-09

**Authors:** Franz Tatzber, Edith Pursch, Ulrike Resch, Roswitha Pfragner, Sandra Holasek, Meinrad Lindschinger, Gerhard Cvirn, Willibald Wonisch

**Affiliations:** ^1^Center of Molecular Medicine, Institute of Pathophysiology and Immunology, Medical University of Graz, Heinrichstrasse 31a, 8010 Graz, Austria; ^2^Institute of Biochemical Engineering, University of Applied Sciences Technikum-Wien, Höchstädtplatz 5-6, 1200 Vienna, Austria; ^3^Department of Vascular Biology and Thrombosis Research, Medical University of Vienna, Schwarzspanierstrasse 17/1, 1090 Vienna, Austria; ^4^Institute of Nutritional and Metabolic Diseases, Outpatient Clinic Laßnitzhöhe, Hauptstrasse 24/Top 21, 8301 Laßnitzhöhe, Austria; ^5^Institute of Physiological Chemistry, Center for Physiological Medicine, Medical University of Graz, Stiftingtalstrasse 6 M1/D/3 8036, Graz, Austria

## Abstract

Oxidatively modified low-density lipoprotein (oLDL) is firmly believed to play an important role in the initiation and development of atherosclerosis, and malonic dialdehyde (MDA) is one of the major lipid peroxidation breakdown products involved in this process. In recent decades, antibodies against MDA-LDL have been detected in human and animal sera. In our study, human B-cells from the peripheral blood of a healthy female donor were fused with the SP2/0 mouse myeloma cell line. Antibody-producing hybridomas were detected by MDA-LDL-IgG/IgM enzyme-linked immunosorbent assays (ELISA) and Cu^++^-oxidized LDL IgG/IgM (oLAb) ELISA. Cells with supernatants emitting positive signals for antibodies were then cloned and after sufficient multiplication frozen and stored under liquid nitrogen. Due to the loss of antibody-producing ability, we established an MDA-LDL-IgM-producing cell line by recloning. This allowed isolation and immortalization of several human B-cells. The human donor had not been immunized with MDA-modified proteins, thus obviously producing MDA-LDL antibodies *in vivo*. Furthermore, using these antibodies for *in vitro* experiments, we were able to demonstrate that MDA epitopes are among the epitopes generated during Cu^++^-LDL oxidation as well. Finally, these antibodies compete in ELISA and cell culture experiments with MDA as a challenging toxin or ligand.

## 1. Introduction

Acute myocardial infarction (AMI) and various malignancies are the most frequent causes of death in Europe and the United States of America (USA), and lipid peroxidation (LPO) due to oxidative stress appears to be importantly involved in various stages of these disease processes. For example, there are many indications that oxidatively modified low-density lipoprotein (oLDL) plays an important role in the initiation and development of atherosclerosis in humans [1, 2]. Obviously, LPO is at least one basic process leading to oLDL by consumption of protective antioxidants present on each low-density lipoprotein (LDL) particle and subsequent initiation of free radical-mediated chain reactions. This leads to formation of reactive oxygen species (ROS) and various highly reactive aldehydes [3, 4], which in turn build up adducts with amino acids of proteins, for example in the case of LDL apolipoprotein B (Apo B). Among all the aldehydes involved in that process, 4-hydroxy-2-nonenal (HNE) and malonic dialdehyde (MDA) have been investigated most extensively [5]. MDA was shown to be significantly increased in smokers [6], in kidney disease [7], and in the course of cardiopulmonary bypass [8], as well as in bipolar disorders [9] and in small-for-gestational age term babies and their mothers [10]. In recent decades, antibodies against oLDL/MDA-LDL have been detected in human and animal sera without or after immunization [11–13]. The presence of such antibodies in human sera strongly indicates that formation of such epitopes followed by specific immune reactions also takes place *in vivo*, presumably as protection against oxidized LDL and atherosclerosis [14, 15], for example, there is an inverse association between MDA-LDL IgM antibodies and carotid atherosclerosis [16]. In 2001, the first human monoclonal antibody was described which recognizes epitopes of oxidized LDL [17, 18]. This group isolated RNA from peripheral mononuclear cells and prepared cDNA to construct a phage display antibody library. After screening of these libraries against MDA-LDL, they were able to isolate a specific antibody binding to oxidized LDL, which recognized apoptotic cells as well. However, the present investigation aimed to isolate and immortalize cells of human origin and so to obtain human monoclonal antibodies for diagnostic and therapeutic purposes as an additional benefit. The successful isolation of cells producing antibodies against LPO products generated *in vitro* so would give further evidence that identical products are also synthesized *in vivo* and cause specific immune reactions. The option of producing human monoclonal antibodies (hMCA) binding to LPO products would further help to minimize anaphylactic reactions in humans if these antibodies were of diagnostic or even therapeutic importance in humans *in vivo*. Finally, using hMCA for diagnostic purposes *in vitro* or in cell cultures would make models possible consisting strictly of human material, so reducing the possibility of unwanted unspecific effects.

## 2. Materials and Methods

### 2.1. Chemicals and Reagents

If not indicated otherwise, all chemicals were obtained from Sigma, St. Louis, USA, and Merck, Darmstadt, Germany. Tissue culture reagents were supplied by Gibco (HAT/HT supplement) and VWR Austria (media, buffers, and fetal bovine serum).

### 2.2. Blood Donation

A healthy human female (68 y) donated 200 ml blood drawn from an antecubital vein and collected into citrate and heparin anticoagulation tubes. The study was conducted in accordance with the guidelines laid out in the Declaration of Helsinki, and all procedures involving human subjects were approved by the Ethics Committee of the Medical University of Graz (28-526 ex 15/16). Written informed consent was obtained from the subject. The donor is a cigarette smoker (<10 cigarettes/day) and did not develop any kind of disease within the next months after venipuncture. Her total lymphocyte count at the time point of blood donation was within the normal range.

### 2.3. Isolation of White Blood Cells (WBC)

A volume of 20 ml Ficoll-Paque solution was pipetted into 50 ml conical tubes, and 20 ml anticoagulated whole blood was carefully layered onto the Ficoll solution. The mixture was then centrifuged at 800 rpm in a Sorvall laboratory centrifuge for 20 minutes. The layer of WBC formed by this procedure was harvested by suction and resuspended in PBS to give 10^8^ cells/ml. This suspension was used for cell fusion within one hour.

### 2.4. Cell Viability and Counts

The number of viable cells was assessed by trypan blue exclusion. Only cell populations with >95% living cells were used in our experiments. Cells were generally counted with improved Neubauer counting chambers.

### 2.5. Cell Fusion

**Figure 1:**
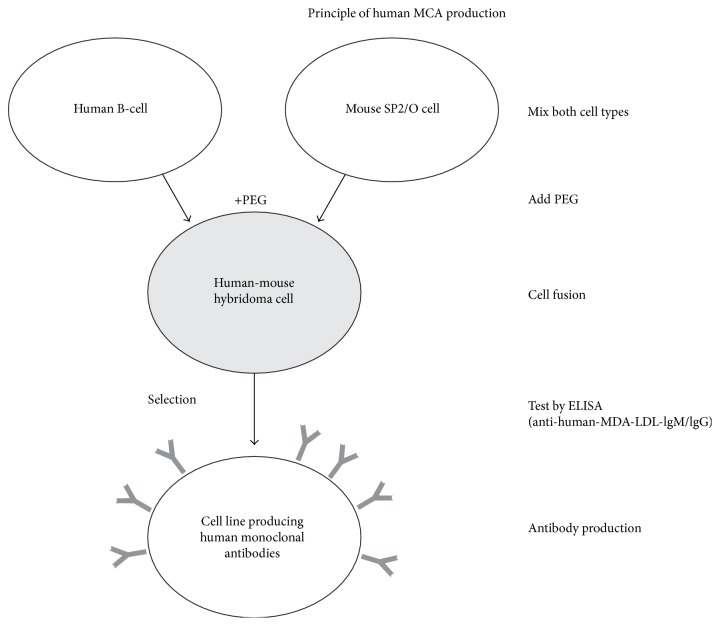
Schematic overview of the process of human monoclonal antibody production. The fusion of human B-cells with murine myeloma cells results in immortalized hybridomas producing human monoclonal antibodies.

Mouse myeloma cells SP2/0 (ATCC number CRL-1581™) were fused with human B-cells following the methods of Köhler and Milstein [19] and Östberg and Pursch [20] with modifications in details. Briefly, human B-cells and mouse myeloma cells SP2/0 were mixed in a ratio of 1 : 10 and centrifuged for 10 minutes at 500 ×g. After removal of the supernatant, the cells were resuspended in 3 ml PEG 400 and cell fusion was allowed for 3 minutes at 37°C. At the end of this period, the 15-fold amount of DMEM was added to the suspension and the cells were again pelleted by centrifugation. The supernatant was removed and the cells were resuspended in DMEM containing 20% fetal bovine serum (FBS). The suspension was finally adjusted to a concentration of 10^5^ cells/ml; 100 *μ*l of this suspension was pipetted into each well of microtitration plates and allowed to recover from the fusion procedure for 24 hours in an incubator at a temperature of 37°C and 5% CO_2_ atmosphere. The scheme for human x mouse cells producing human IgG/IgM antibodies against oLDL is shown in Figure 1.

### 2.6. HAT Selection

To eliminate unfused and fused cells without the participation of human B-cells, 100 *μ*l DMEM containing hypoxanthine/aminopterin/thymidine (HAT) supplement diluted according to manufacturer's instructions (1 : 50) was added to each well and incubated for 48 hours. At the end of this period, 100 ml of the medium was removed and replaced by DMEM containing 10% FBS further addressed as clone medium. This procedure was repeated twice and the aminopterin concentration thus continuously reduced over six days, while hypoxanthine and thymidine were supplemented following manufacturer's recommendations (1 : 100). Finally, the entire medium was removed from the wells by suction and replaced by clone medium.

### 2.7. Identification of Productive Clones

At the end of the HAT selection period, supernatants of each well were tested for antibodies binding to Cu^++^-oxidized LDL and/or MDA-LDL with commercially available ELISA methods (oLAb-ELISA, Biomedica, Vienna, Austria; MDA-LDL IgG/IgM, LDN Ltd., Nordhorn, Germany) adapted for the purposes of this investigation [21]. In detail, 10 *μ*l medium of each well was transferred into wells of ELISA plates coated with Cu^++^-oxidized and MDA-LDL. Then, 200 *μ*l assay buffer (PBS containing 1% BSA) was added and the plates were incubated at 37°C for 2 hours. The plates were washed 4 times with a solution containing PBS plus 0.2% Tween 20 and dried on cellulose; then, IgG and IgM horseradish peroxidase (HRP) conjugates for detection of human IgG and IgM antibodies were diluted according to manufacturer's recommendations, and 100 *μ*l of diluted conjugate was added to each well. The ELISA plates were then incubated for 40 ± 5 minutes at room temperature (20 ± 1°C) followed by another washing step. For development, 100 *μ*l of a citrate/phosphate buffer (pH = 5.0) containing tetramethylbenzidine (TMB) was added to each well; after 20 minutes, the colour reaction was stopped by the addition of 50 *μ*l of 2 N H_2_SO_4_. Absorptions were measured photometrically on a microplate reader at a wavelength of 450 nm with 650 nm as reference wavelength [22]. Samples that gave signals exceeding those of blanks (mean_blank_ + 3 s) were considered positive. Cells from such wells were then transferred to 24-well plates and allowed to grow until they reached confluency. Clones that remained positive after repeated testing were transferred to larger 6-well plates and tissue culture bottles of 25 cm^2^ and 75 cm^2^. Those clones that remained positive over all passages were subsequently frozen and kept under liquid nitrogen until further use.

### 2.8. Freezing of Clones

Prior to freezing, cells were passaged into 75 cm^2^ tissue culture bottles and grown until they reached a coverage/confluency level of >80%, that is, at least 80% of the bottom surface of the flasks was covered with cells. The cells by then had reached an exponential growth phase and were harvested mechanically by vigorous shaking of the bottles, which caused >90% of the cells to detach from the solid phase. After centrifugation at 800 ×g for 10 minutes, the supernatant was discarded and the cells were resuspended in freeze medium (DMEM + 20% FBS + 10% DMSO (dimethylsulfoxide)) to give a concentration of 5 × 10^6^ cells/ml. Fractions of 1 ml volume were then filled into Nunc cryotubes (Nunc Inc., Denmark) that were tightly screwed down, gradually cooled to −80°C, and finally stored under liquid nitrogen.

### 2.9. Recloning/Subcloning

After the cells were thawed, the human/mouse hybridomas were seeded into a 75 cm^2^ bottle and allowed to grow to 80% confluency. The cells were harvested as described above and seeded into 96-well plates at concentrations of 5 cells/ml to give 1 cell/well. After the development of clones, the supernatants were tested for monoclonal human IgM antibodies by ELISA.

### 2.10. Competition Experiments

MDA-LDL and Cu^++^-oxidized LDL-coated ELISA plates were allowed to react with either clone 3G5 or subclone 3G5/D4 IgM antibodies. Various LDL preparations and antioxidants were added to the reaction mixture in serial dilutions. Assay and incubation scheme remained as previously described [22].

### 2.11. Statistics

Statistical analysis (*t*-test and linear correlation) was performed using the Sigma-Stat and Sigma-Plot package (SPSS, Erkrath, Germany).

## 3. Results

### 3.1. Cloning Experiments

After the clones were first tested by ELISA on day 7, 116 out of 480 wells were identified as giving positive signals for human IgG antibodies binding to oLDL. Only 45 wells contained cells producing human IgM antibodies. On day 52, after six further passages of the cells in formerly positive wells, all the IgG clones and 89% of the IgM clones had stopped producing antibodies. The remaining 5 clones producing human anti-oLDL IgM antibodies were frozen and stored in liquid nitrogen. After 20 more days, only 2 clones with stable production of human monoclonal IgM antibodies remained; these were again frozen and used to produce antibodies in their supernatants, which were stored at 4°C under aseptic conditions (Table 1).

**Figure 2:**
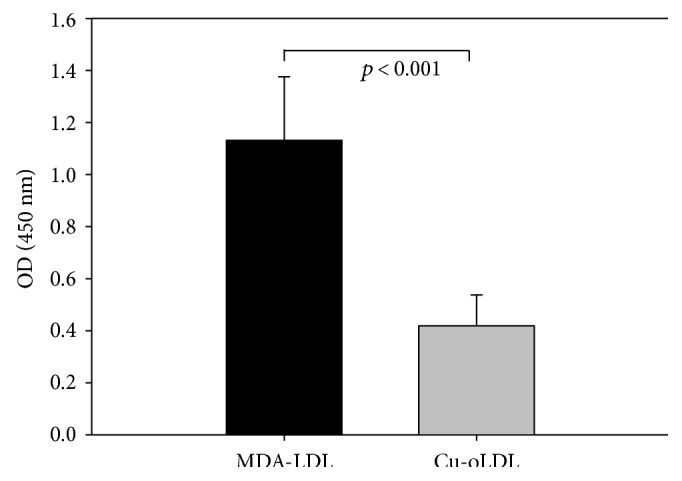
Signals of the MCA-producing IgM clone 3G5 on plates coated with MDA-LDL and Cu^++^-oxidized LDL. The weaker binding on Cu^++^-oxidized LDL-coated plates (*p* < 0.001) indicates that MDA is also produced by copper oxidation, but due to several other modifications, less appropriate binding sites occur for the MCA.

The signals produced by clone 3G5, which was used for further investigations, were more pronounced on MDA-LDL-coated plates than on Cu^++^-oxidized LDL-coated plates (Figure 2).

**Figure 3:**
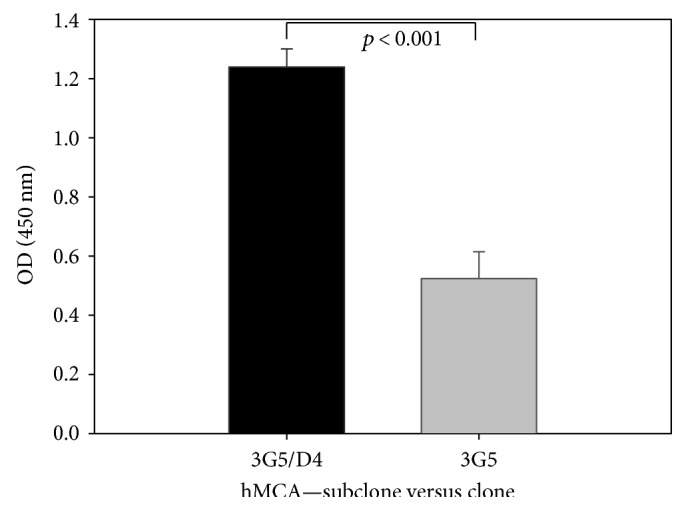
Signals of MCA-IgM antibodies on MDA-coated plates. Reclonation increased the IgM production in the subclone significantly (*p* < 0.001) at least of 100% compared to the clone.

**Figure 4:**
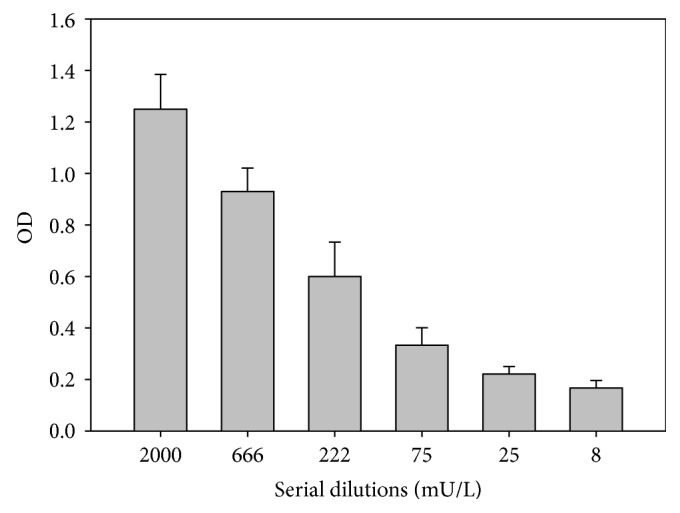
Results of serial dilutions of anti-MDA-LDL IgM MCA 3G5 on MDA-LDL coated plates. A signal reduction of 50% of the highest signal requires a dilution of approximately a factor 10.

After subcloning the cells of clone 3G5, the results were similar to those from the initial cloning. The initial number of identified subclones (*n* = 56) was reduced to 7 after 10 further passages; these were frozen and kept under liquid nitrogen. Comparison of signals of clone 3G5 and subclone 3G5/D4 showed at least doubling of absorptions in supernatants of the subclone (Figure 3). Dilution experiments of the subclone showed that a 10-fold dilution was necessary to reduce absorption by 50% (Figure 4), indicating that the human IgM antibody production of the subclone was increased by a factor of 10 compared with the initial clone.

**Figure 5:**
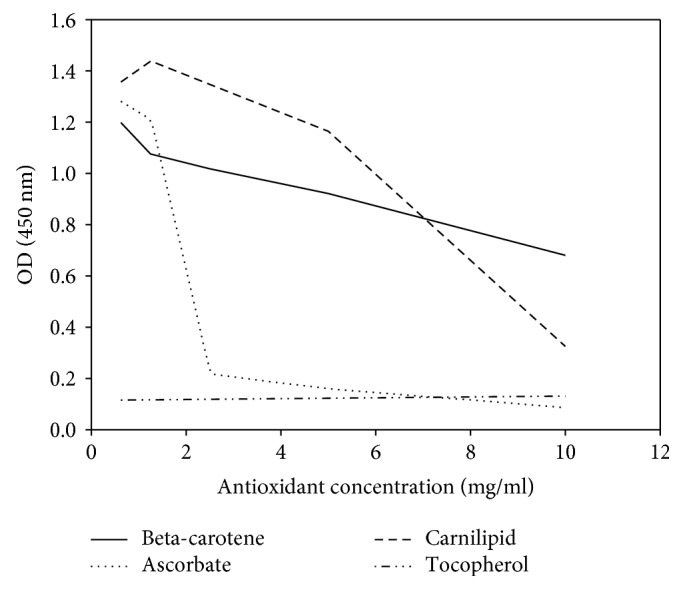
The effect of several antioxidants used as competitors in the monoclonal anti-MDA-LDL IgM ELISA. Aldehyde residues of MDA were reduced, presumably to alcohols, by antioxidants, thus precluding binding of the MCA.

Competition experiments with other adducts of LDL than MDA or Cu^++^-oxidized LDL like hexanal-LDL, 4-HNE-LDL, or HOCl-LDL did not show these substances to interfere with the antibody-antigen reaction of the MDA-LDL plate. Thus, other aldehydes than MDA did not lead to signal reductions. On the other hand, addition of reducing substances like antioxidants to the reaction mixture produced a dose-dependent reduction of absorption signals, indicating that the antigen-antibody reaction was inhibited by reducing substances such as antioxidants (Figure 5).

## 4. Discussion

This study describes the isolation and immortalization of human cells producing antibodies to MDA and Cu^++^-oxidized LDL. This was achieved by fusion of human B-cells with the SP2/0 murine myeloma cell line. As expected, most of the human-mouse fusions did not result in permanently productive hybridoma cells. All the cells secreting IgG antibodies could not be immortalized with stable production of these molecules. Only 2 cell lines that produced human anti-MDA-LDL-IgM remained stable enough to be frozen in an antibody-producing state. As the production of antibodies was reduced after thawing, we decided to reclone the antibody-producing cell line. This subcloning experiment resulted in seven stable subclones, which secreted approximately the 10-fold concentration of IgM antibodies binding to MDA-LDL compared to the mother clone and remained stable until freezing after six further passages.

From these data, we conclude that the fusion of murine and human cells is possible and at least in individual cases can result in stable secretion of human antibodies binding to oLDL. Furthermore, as the human donor did not receive any kind of immunization against MDA-LDL, these results support the hypothesis that epitopes like MDA-LDL adducts are generated *in vivo* and may cause immune reactions leading to anti-MDA-LDL immunoglobulins. It was reported previously that MDA epitopes were predominantly found on circulating microparticles released from apoptotic and activated cells as physiological carriers of oxidation-specific epitopes. The stimulation of THP-1 cells and primary monocytes through these oxidation-specific epitopes, which produce interleukin 8, could be inhibited by MDA-LDL IgM antibodies [23]. This is in accordance with the findings of Rahman et al. [24], indicating high MDA-IgM antibody titers as a striking protection marker for atherosclerosis in systemic lupus erythematosus patients, increasing even the uptake of apoptotic cells. The protective mechanism of increased MDA-LDL IgM antibody titers is also supported in children with familial hypercholesterolemia [25] and reflects oxidative modifications that occur in systemic diseases [26].

The fact that these IgM antibodies gave stronger signals on MDA-LDL-coated plates compared to Cu^++^-oxidized LDL indicates that MDA is also produced during Cu^++^-LDL oxidation, but together with several other LPO products that the antibody does not react with, so leading to lower absorptions on Cu^++^-oxidized LDL-coated plates. In this respect, it should be noted that MDA-LDL IgM and copper oxidized LDL antibody titers decrease simultaneously after percutaneous coronary intervention and a subsequent increase of IgM antibodies persisted for one month and up to six months for IgG antibodies [27].

The specificity of these IgM antibodies secreted by the immortalized human x mouse hybridomas is not only supported by the fact that only MDA-LDL and Cu^++^ oxidized-LDL can inhibit the immune reaction between antibodies and MDA-LDL bound to the solid phase but also by the finding that reducing substances such as antioxidants can prevent this immune reaction in a dose-dependent manner. As MDA is a molecule with two aldehyde residues, it reacts when one aldehyde group forms adducts to amino acids, while the other remains unchanged if it is not used for crosslinking. In the presence of antioxidants such as ascorbic acid or alpha-tocopherol, Schiff bases or even alcohols may be formed, which the monoclonal MDA-LDL-IgM antibody does not recognize. This finding corresponds to the antioxidant effect of a phytonutrient supplementation showing a significant decrease of MDA in smokers [6]. Conversely, a decrease in vitamin C increased plasma MDA in cystic fibrosis patients [28].

Finally, competition or tissue culture models of atherosclerosis could be applied in screening for antioxidant/antiatherosclerotic substances or mixtures, which might contribute both to further understanding and even to therapeutic concepts of atherosclerosis and myocardial infarction.

## Figures and Tables

**Table 1:** Decrease of the number of monoclonal antibodies producing clones during the observation period. It should be noted that all IgG clones stopped MCA production and only 2 IgM-producing clones survived during the observation period.

Time (d)	hMCA IgG	hMCA IgM
7	121	45
17	16	12
52	0	5
75	0	2

## Abbreviations

4-HNE: 4-Hydroxynonenal
Cu: Copper
DMEM: Dulbecco's modified minimal essential medium
DMSO: Dimethyl sulfoxide
ELISA: Enzyme-linked immunosorbent assay
FBS: Fetal bovine serum
HOCl: Hypochlorite
IgG: Immunoglobulin G
IgM: Immunoglobulin M
LDL: Low-density lipoprotein
LPO: Lipid peroxidation
MDA: Malonic dialdehyde
PBS: Phosphate-buffered saline
oLDL: Oxidized low-density lipoprotein.
